# Association between Levels of Urine Di-(2-ethylhexyl)phthalate Metabolites and Heart Rate Variability in Young Adults

**DOI:** 10.3390/toxics9120351

**Published:** 2021-12-12

**Authors:** Ching-Way Chen, Shu-Yu Tang, Jin-Shiang Hwang, Chang-Chuan Chan, Cheng-Chih Hsu, Chien-Yu Lin, Ta-Chen Su

**Affiliations:** 1 Department of Internal Medicine, National Taiwan University Hospital Yunlin Branch, Yunlin 640203, Taiwan; brendon32402@gmail.com (C.-W.C.); boryangcafe@gmail.com (S.-Y.T.); 2 Institute of Statistical Science, Academia Sinica, Taipei 11529, Taiwan; jshwang@stat.sinica.edu.tw; 3 Institute of Environmental and Occupational Health Sciences, College of Public Health, National Taiwan University, Taipei 10017, Taiwan; ccchan@ntu.edu.tw; 4 Department of Chemistry, National Taiwan University, Taipei 10017, Taiwan; ccrhsu@ntu.edu.tw; 5 Department of Internal Medicine, En Chu Kong Hospital, New Taipei City 237414, Taiwan; lin7010@mail2000.com.tw; 6 Department of Environmental and Occupational Medicine, National Taiwan University Hospital, Taipei 10002, Taiwan; 7 Division of Cardiology, Department of Internal Medicine, National Taiwan University Hospital, Taipei 10002, Taiwan

**Keywords:** plasticizers, di-(2-ethylhexyl) phthalate, health assessment, heart rate variability, young adults

## Abstract

Phthalate exposure is associated with cardiovascular risk. Among the various phthalates, di-(2-ethylhexyl) phthalate (DEHP) is a deleterious plasticizer in our daily lives. This study investigated the association between DEHP exposure and the alteration of heart rate variability (HRV). During 2017–2019, we recruited 974 young adults to investigate the effects of living environments and dietary habits on cardiometabolic disorders in Taiwan. We quantitatively analyzed urinary metabolites of DHEP. A continuous electrocardiogram was recorded to obtain a 5-min ECG. Time-domain and frequency-domain HRV analyses were performed. Multiple linear regression showed that urinary oxidized DEHP metabolites MEHHP and MEOHP were associated with decreased HRV after controlling for associated cardiovascular risk factors. A higher MEHHP level was associated with a lower triangular interpolation of NN interval histogram (TINN), very low frequency (VLF), and low frequency/high frequency (LF/HF) ratio. A higher MEOHP level was associated with a decreased LF/HF ratio. In addition, trend analysis showed that higher MEHHP and MEOHP quantiles were significantly associated with a decreased LF/HF ratio. DEHP is a potentially harmful and invisible chemical. The urinary DEHP metabolites MEHHP and MEOHP are associated with decreased HRV, indicating an adverse effect on autonomic balance in young adults in Taiwan.

## 1. Introduction

Phthalate esters are colorless and odorless chemicals widely used as plasticizers to add flexibility and resilience to plastic products [1]. Phthalate esters are essential to make cosmetics, medical devices, plastic, and rubber products. They are hydrophobic and bound to polymers with noncovalent bonding and, thus, readily leach into their environment. These chemicals are invisible chemicals and are hard to detect. Human exposure is possible through dietary ingestion, air inhalation, or direct contact. Among various phthalate esters, di-(2-ethylhexyl) phthalate (DEHP) is a widely used plasticizer we encounter in daily life [2].

After entering the human body, DEHP is rapidly degraded to its monoester, mono-(2-ethylhexyl) phthalate (MEHP), which is further metabolized by various hydroxylation and oxidation reactions [3,4,5]. Two of the major secondary oxidized DEHP metabolites are mono-(2-ethyl-5-oxohexyl) phthalate (MEOHP) and mono-(2-ethyl-5-hydroxyhexyl) phthalate (MEHHP) [6]. DEHP is eliminated from the body in the form of its metabolites via urine. Although phthalate excretion is rapid, with a half-life of less than 24 h, continuous daily exposure and ingestion of phthalates still cause persistent physiological effects due to steady concentrations in the human body.

Exposure to DEHP is associated with cardiovascular risk factors, such as increased blood pressure [7], insulin resistance [8,9], and diabetes mellitus [10]. The exposure is also associated with increased inflammation markers of absolute neutrophil counts, alkaline phosphatase, ferritin levels, and C-reactive proteins [11,12]. Our previous studies demonstrated that urinary phthalate metabolites were associated with apoptotic microparticles from endothelial cells and platelets, insulin resistance, and subclinical atherosclerosis in terms of increased carotid intima–media thickness [13,14]. Urinary phthalates have been associated with a higher risk of stroke in the U.S. [15], and we have also demonstrated that increased DEHP exposure may be linked to patients with coronary heart diseases [16]. DEHP accelerates atherosclerosis by disturbing cholesterol homeostasis, and the inflammatory response has been ascribed to a pathogenic mechanism in animal models [17]. Our recent study also confirmed that the global DNA methylation marker 5 mdC/dG may mediate the association between DEHP exposure and subclinical atherosclerosis [18]. However, these attributable risk factors could not fully explain the underlying adverse effects of DEHP on the cardiac and cardiovascular systems [16,19].

Heart rate variability (HRV) measures the fluctuation of the heart rate. HRV is a noninvasive method to analyze autonomic nervous system effects. For the general population, decreasing heart rate variability is associated with an increased incidence of cardiac events [20] and mortality [21,22]. Decreased HRV was associated with coronary artery disease [23], heart failure [24], pulmonary hypertension [25], and impaired renal function [26]. For patients with congestive heart failure [27] or after myocardial infarction [28,29,30], decreased HRV is associated with a higher mortality rate. All this evidence suggests that HRV is a useful tool to evaluate autonomic dysfunction and cardiac adverse effects. In animal models, DEHP-treated mice had decreased HRV, enhanced cardiovascular reactivity, and prolonged blood pressure recovery [31]. This animal model suggested that DEHP may cause adverse cardiac effects through the mechanism of decreasing HRV due to autonomic dysfunction.

However, the association between DEHP exposure and HRV in humans is still unclear. Traditional cardiovascular risk factors are possible confounding factors for both DEHP exposure and HRV. Therefore, we focused on a young population in this study to investigate the association.

## 2. Materials and Methods

### 2.1. Subjects

**Figure 1:**
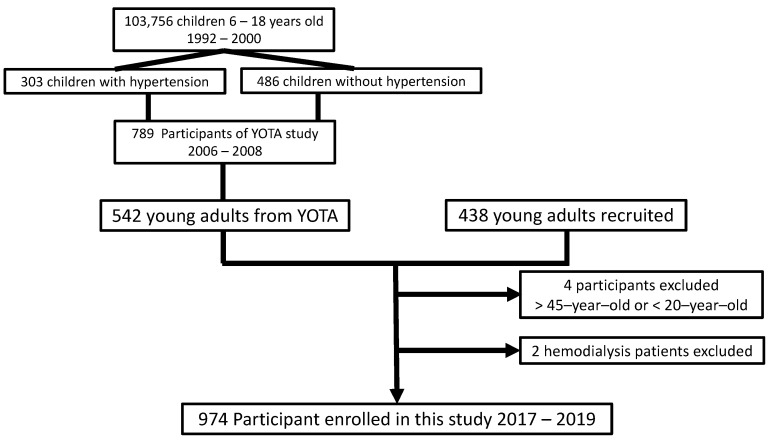
Recruitment flow chart for the New YOTA study.

From 1992 to 2000, a nationwide urine screening program for early renal disease was conducted for Taiwanese children between 6 and 18 years of age [32]. Among over 103,756 students who underwent the screening, 303 children with hypertension and 486 children with normal blood pressure living in the Taipei area joined the Young Taiwan cohort (YOTA) study between 2006 and 2008 under informed consent and parental agreements in National Taiwan University Hospital [13,14]. During 2017–2019, we recruited 980 young adults to investigate the effects of living environments and dietary habits on cardiometabolic disorders in Taipei, Taiwan. There were 542 YOTA participants, and 438 young adults of similar age were recruited as the New YOTA cohorts. The New YOTA cohort was more comprehensive in representing the young adults with low cardiovascular risk factors in Taiwan, since more than half of the participants had been followed up for more than 10 years. The other 438 participants were also recruited under the same screening protocols. Among all 980 participants, 4 participants were excluded due to being less than 20 years old or more than 45 years old. Two participants were excluded from the New YOTA study due to no measurable urine sample under hemodialysis status. Thus, 974 participants were enrolled in this study (Figure 1).

### 2.2. Assessment of Clinical Information and Risk Stratification

Basic cardiovascular risk factors, including age, sex, weight, height, smoking habits, alcohol drinking, and exercise habits, and the personal living environment characteristics and dietary habits of each participant were collected. Exercise habits included exercise form, content, frequency, and duration of each section. Living environmental characteristics included ventilation, house plants, incense burning, and indoor air quality. Dietary habits included content, calories, and the ratio of high-fiber diets. The arterial pressure waveform was recorded by a cuff sphygmomanometer using an oscillometric BP device (DynaPulse 200 M, Pulse Metric Inc., San Diego, CA, USA) [33]. The arterial pressure waveform was measured from left and right hands after at least 5 min of rest in a sitting position in a quiet room. Hypertension was diagnosed if the mean systolic blood pressure was greater than 140 mmHg, diastolic pressure was greater than 90 mmHg, or the patient was taking antihypertension medications.

Blood samples were obtained via the antecubital vein of each participant after overnight fasting for 10–14 h. The serum cholesterol, triglyceride, and low- and high-density lipoprotein cholesterol (LDL-C and HDL-C, respectively) levels and plasma glucose were measured using an autoanalyzer (Toshiba, TBA-200FR; Toshiba, Tokyo, Japan). Biochemical examination for each participant was performed according to standard lab protocols/methods. Diabetes mellitus was diagnosed according to the American Diabetes Association criteria, and those whose fasting glucose levels were equal to or greater than 126 mg/dL (7.0 mmol/L) had diabetes. We measured the weight and height of the participants by standard methods. Body mass index (BMI) was calculated using weight (in kilograms) divided by the square of the height (in meters).

### 2.3. Urinary Phthalates Metabolites

First-voiding morning urine was collected from every participant between 6 and 8 a.m. to analyze urinary metabolites of phthalates, including MEHP, MEHHP, and MEOHP. The sample processing for urinary phthalate metabolites was described in detail in our previous reports [5,9,14]. Finally, urine mixtures were quantitatively analyzed by liquid chromatography with a tandem mass spectrometric (LC-MS/MS) system.

Regarding the quality assurance and control of DEHP metabolites, we prepared blank samples for each batch of samples during sample preparation. Internal quality control was performed using pooled quality control urine samples, with precision ranging from 6% to 26%, depending on the metabolite. Alongside pooled urine samples for each batch, low-concentration (20 mg/L) and high-concentration (50 mg/L) quality control materials were also analyzed. The method detection limits of MEHP, MEHHP, and MEOHP were 0.7, 0.1, and 0.1 μg/L, respectively. External quality assurance was assessed using the German External Quality Assessment Scheme for Biological Monitoring (G-EQUAS) [14].

### 2.4. Heart Rate Variability Analysis

After a 15 min rest on the same day of urine and blood sample collection, we performed the resting electrocardiogram (ECG) examination in the supine position during the daytime (9:00 a.m. to 12:00 p.m.) for each participant using an HRV+ (BeneGear, Taipei, Taiwan) with a sampling rate of 250 Hz (4 ms). A complete 5-min segment of the N–N interval was taken for HRV analysis.

Time-domain and frequency-domain analyses were analyzed for heart rate variability. All analyses were performed according to the recommendations of the European Society of Cardiology and the North American Society of Pacing and Electrophysiology [34]. The time-domain measurements of HRV included the mean of the R–R intervals, the standard deviation of the normal-to-normal intervals (SDNN), root mean square successive differences (RMSSDs), between adjacent normal-to-normal intervals and percentage of absolute differences in normal RR intervals greater than 50 ms (pNN50), and triangular interpolation of the NN interval histogram (TINN), indicating the baseline width of the RR interval histogram. The frequency-domain measurements of HRV included very low frequency (VLF, <0.04 Hz), low frequency (LF: 0.04–0.15 Hz), high frequency (HF: 0.15–0.40 Hz), and LF/HF ratio, which were calculated by Welch’s averaged periodogram of the normal-to-normal intervals. These parameters represented the modulation of sympathetic and parasympathetic activity to heart rate variability. The details of translating ECG wave complexes to HRV indices are given in our previous study [35].

### 2.5. Statistical Analysis

We performed statistical analysis using IBM SPSS Statistics for Windows, Version 26.0. (IBM Corp., Armonk, NY, US). Three urine DEHP metabolites (MEHP, MEHHP, and MEOHP) were log-transformed to fit a normal distribution, as confirmed by the Kolmogorov–Smirnov test. Samples of urine phthalate metabolites below the detection limit were recorded as the detection limit divided by the square root of 2. The concentration of urine phthalate metabolites was calibrated by urine creatinine. Phthalate metabolite concentrations are expressed as the mean ± standard deviation. The Student’s *t*-test was performed to compare the concentrations of urinary phthalate metabolites in different subgroups. The HRV parameters following a non-normal distribution were also log-transformed. A multiple linear regression model was used to evaluate the dose–response relationships between HRV parameters and urine phthalate metabolites. To adjust the effects of possible confounders, covariates of age, sex, BMI z-score, systolic BP, fasting blood sugar, and LDL-C were added into the multiple linear regression model. The estimated coefficient of urinary phthalate metabolites and its 95% confidence interval (95% CI) were calculated to measure the effect of a 1-unit increase in phthalate metabolites to HRV parameters after adjusting the covariates. We considered an estimate was statistically significant when the *p* value was less than 0.05. To confirm the association between the concentration of urinary phthalate metabolites and HRV parameters, we divided the participants into four exposure groups according to the quantiles of phthalate metabolite concentrations. Trend analysis in one-way analysis of variance (ANOVA) was performed to test the linear trend of HRV parameters among the four exposure groups.

## 3. Results

### 3.1. Participant Characteristics

Among the 974 participants recruited in this study, there were 407 men and 567 women. The mean age was 31.9 years old. The geometric means and standard deviations of the creatinine-adjusted urinary DEHP metabolites of different characteristic subgroups are listed in Table 1. Women had statistically significantly higher levels of MEHHP and MEOHP than men. Participants aged between 18 and 32 had a higher level of MEHP and a lower level of MEOHP. Participants with normal LDL-C had a higher level of MEOHP. Participants with higher education had significantly lower levels of MEHHP and MEOHP. Other clinical characteristics, such as hypertension, diabetes mellitus, body mass index, and current smoking had no significant association with urinary DEHP metabolites (Table 1).

### 3.2. Heart Rate Variability Analysis

Univariable analysis of the time domain showed that higher levels of MEHHP and MEOHP were associated with decreased TINN (Table 2). Frequency domain analysis revealed that higher levels of MEHHP and MEOHP were associated with a lower VLF, LF, and LF/HF ratio, as shown in Table 3. The molecular summation of DEHP metabolites (ΣDEHP metabolites) showed a similar trend, though not statistically significant, except for TINN (*p* = 0.044) (Table 2).

Univariable analysis of HRV showed that older age; being a woman; higher BMI z-score; and higher BP, blood sugar, and LDL-C were negatively associated with time-domain HRV in the lower mean RR interval, SDNN, RMSSD, pNN50, and TINN. In addition, we also showed a negative association between cardiovascular risk factors and frequency-domain HRV for the VLF, LF, HF, and the LF/HF ratio (Tables S1 and S2).

### 3.3. Multiple Linear Regression Models

In the multiple linear regression analysis of the HRV parameters after adjusting for age, sex, BMI z-score, blood pressure, fasting blood glucose, and LDL-C, we demonstrated that a higher MEHHP level was associated with a significantly lower TINN, with an estimated coefficient of −0.057 and 95% CI = (−0.102~−0.011), VLF, with −0.153 (−0.210~−0.010), and LF/HF ratio, −0.110 (−0.200~−0.020) (Table 4). A higher MEOHP level was associated with a significant decrease in LF/HF ratio, with an estimated coefficient of −0.101 (−0.184~−0.019) (Table 5). The molecular summation of DEHP metabolites (ΣDEHP) did not reveal similar associations.

**Figure 2:**
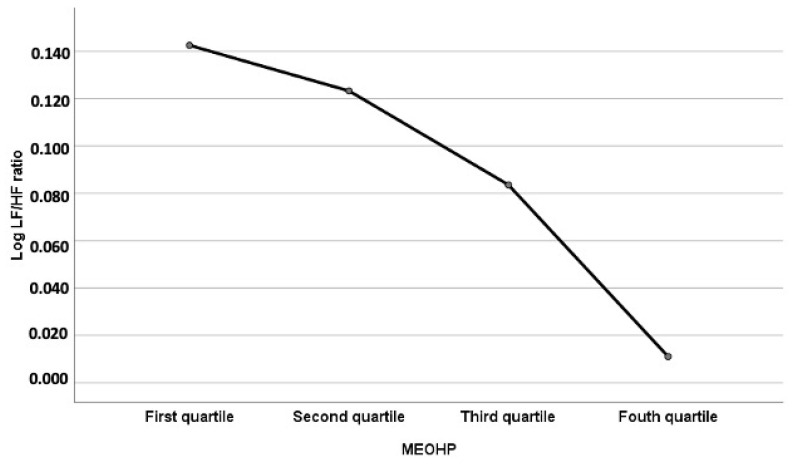
Means plot of log LF/HF ratio among four exposure groups categorized by the four quartiles of MEOHP measurements. Abbreviations: MEOHP: mono-(2-ethyl-5-oxohexyl) phthalate, LF: low frequency, HF: high frequency.

The trend analysis in one-way ANOVA showed that exposure to higher MEHHP and MEOHP quantiles were significantly associated with a lower LF/HF ratio. The *p* values for trend were 0.014 for MEHHP and 0.001 for MEOHP (Table 6 and Figure 2).

## 4. Discussion

The most important finding of this study is that urinary phthalate metabolites were associated with decreased HRV in young adults. This is the first report to show an association between DEHP exposure and decreased autonomic balance in a human study.

Previous studies have shown that phthalate DEHP exposure alters the autonomic nervous system and decreases HRV in animal models [31]. DEHP exposure also increased the expression of the genes encoding endothelin-1 and angiotensin-converting enzyme in heart tissue [31]. Other studies showed that DEHP-treated cardiomyocytes had increased gene expression of calcium-handling genes [36] and subsequently markedly reduced cardiac network synchronicity of DEHP-treated cardiomyocytes [37]. These were possible mechanisms of decreased HRV caused by DEHP exposure.

Endothelial function was associated with heart rate variability in animal studies [38]. Endothelial dysfunction was also associated with decreased HRV in healthy adults and patients with stable coronary artery disease or diabetes mellitus [39,40,41]. Increased carotid intima–media thickness was associated with decreased HRV in previous studies [42,43]. Our previous studies showed that urinary phthalate metabolites were associated with endothelial dysfunction, insulin resistance, and increased carotid intima-media thickness [13,14,44]. Thus, exposure to DEHP may mediate impaired HRV through these mechanisms.

Our study showed that higher concentrations of urinary MEHHP and MEOHP were associated with decreased HRV, primarily in frequency domain HRV. Both MEHHP and MEOHP were two of the major secondary and oxidized urinary metabolites of DEHP. We did not identify this association in MEHP, which is the monoester of DEHP after the first step of hydrolysis metabolism. The possible explanations for the results of our study are as follows. First, the formation of MEHP from DEHP is possible through abiotic hydrolysis during urinary sample collection, storage, and processing. MEHHP and MEOHP are secondary oxidized DEHP metabolites in the liver, and thus are less likely to be contaminated during sample handling [6]. Second, urinary MEHP accounts for less than 10% of DEHP intake [45]. Urinary MEHP also has the shortest half-life (approximately 5 h) compared to urinary MEHHP or MEOHP (both approximately 10 h) [4]. Urinary MEHP could thus be more easily influenced by prolonged sample handling during urine collection at the study site. For example, it took approximately 6–8 h before urine samples were sent to the refrigerator to freeze in this study.

The molecular summation of DEHP metabolites also did not show this association with decreased HRV. In addition to the possible explanations above, another reason may be that we did not measure all urinary metabolites in this study. Four metabolites have been suggested to measure in the National Report on Human Exposure to Environmental Chemicals: MEHP, MEOHP, MEHHP, and mono-(2-ethyl-5-carboxypentyl) phthalate (MECPP) [46]. Although the significance of each unchecked metabolite may be low, the potential health effects of unchecked metabolites are still uncertain. Thus, urinary MEHP and the molecular summation of DEHP metabolites might not be able to reflect the actual DEHP exposure and its pathophysiological effects on autonomic dysfunction.

HRV analysis includes time-domain analysis, frequency-domain analysis, and nonlinear regression. We used time-domain and frequency-domain HRV analysis in our study, as studies showed that linear and nonlinear HRV analyses yielded similar conclusions [47,48]. Multivariable linear regression was performed to control for the possible confounding factors, such as age, sex, BMI z-score, SBP, fasting glucose, and LDL. Thus, the association between decreased HRV and urinary DEHP oxidized metabolites was less likely due to other confounding factors.

This study has many strengths. First, most cardiovascular risk factors, such as older age; a higher BMI z-score; and higher BP, blood sugar, and LDL-C, were mostly associated with lower time-domain and frequency-domain HRV, which also corroborated the reliable measurement of HRV. Second, measurements of phthalate metabolites are compatible with our previous study in which women had higher levels of DEHP metabolites than men [14]. Third, even after controlling for most associated confounders, DEHP metabolites were still strongly linked to reduced HRV parameters. Fourth, decreased HRV was noted in various physiological and pathophysiological statuses, such as major cardiovascular risk factors. This evidence also corroborates the validity of HRV measurements. We recruited young adults with low cardiovascular risk and prevented these possible confounding factors.

This study also has several limitations. First, this was a cross-sectional study, as we initiated a new Young Taiwan cohort (YOTA) study. A previous study showed a temporal trend of decreased urinary phthalate concentrations 5 years after the regulation of phthalates [49]. Even though this is the first study reporting decreased HRV associated with DEHP exposure, a future follow-up is warranted to confirm the temporal trend and causality of HRV alterations due to phthalate exposure. Second, our cohort was recruited between 2017 and 2019. Both the urine sampling for phthalates exposure and ECG for heart rate variability analysis for all the participants were taken simultaneously while receiving cardiovascular health examination. There was no major change for phthalates regulations during 2017–2019. Thus, we did not take the year of data collection as a confounder during statistical analysis. Third, the use of antihypertensive drugs is one of the possible routes of phthalate exposure. Although only 4.2 percent of participants in this study fit the definition of hypertension and most of them did not take any antihypertensive medications, the use of antihypertensive drugs could still be a potential confounder. Finally, a single measurement of first-morning spot urine may not represent the real phthalate exposure of each participant. We could not confirm whether this single measurement could represent the steady state of the daily phthalate exposure for each individual. Repeated measurement studies for measurement reliability and consistency are needed in the future.

## 5. Conclusions

DEHP is a potentially harmful and invisible chemical. Urinary DEHP oxidized metabolites MEHHP and MEOHP are associated with decreased HRV in time-domain and frequency-domain analysis. Preventing phthalate exposure, particularly DEHP, could prevent environmental pollution and minimize its cardiovascular hazards to humans. A prospective cohort study is warranted to confirm the causality of DEHP exposure and decreased HRV.

## Figures and Tables

**Table 1:** Basic demographics and subgroup analysis of urinary phthalate metabolites.

	Log MEHP (μg/g Creatinine)			Log MEHHP (μg/g Creatinine)		Log MEOHP (μg/g Creatinine)	
	N (%)	Mean (SD)	*p*-Value	Mean (SD)	*p*-Value	Mean (SD)	*p*-Value
Total	974 (100)	0.682 (0.693)		0.783 (0.296)		−0.386 (0.327)	
Median (IQR) *		0.553 (0.943) *		0.774 (0.376) *		−0.395 (0.407) *	
Sex			0.193		0.001		< 0.001
Male	407 (41.8)	0.648 (0.709)		0.747 (0.297)		−0.448 (0.332)	
Female	567 (58.2)	0.707 (0.681)		0.809 (0.293)		−0.341 (0.316)	
Age			0.040		0.303		0.048
18–32	453 (46.5)	0.725 (0.720)		0.773 (0.281)		−0.408 (0.320)	
>32	521 (53.5)	0.634 (0.659)		0.792 (0.309)		−0.367 (0.332)	
Hypertension			0.097		0.132		0.314
Yes	41 (4.2)	0.916 (0.905)		0.858 (0.317)		−0.329 (0.365)	
No	933 (95.8)	0.673 (0.681)		0.780 (0.295)		−0.388 (0.325)	
Diabetes mellitus			0.536		0.102		0.290
Yes	30 (3.1)	0.759 (0.674)		0.858 (0.245)		−0.337 (0.252)	
No	944 (96.9)	0.680 (0.694)		0.781 (0.298)		−0.388 (0.329)	
LDL-C, mg/dL			0.532		0.084		0.010
≥130	273 (28)	0.660 (0.736)		0.757 (0.294)		−0.429 (0.324)	
<130	701 (72)	0.692 (0.676)		0.794 (0.297)		−0.369 (0.124)	
BMI Z-score			0.740		0.789		0.613
≥−0.20	511 (52.5)	0.676 (0.718)		0.781 (0.309)		−0.391 (0.344)	
<−0.20	463 (47.5)	0.690 (0.665)		0.786 (0.281)		−0.380 (0.307)	
Smoking habit			0.513		0.270		0.089
Smoker	177 (18.2)	0.652 (0.682)		0.761 (0.299)		−0.426 (0.345)	
Nonsmoker	797 (81.8)	0.689 (0.696)		0.788 (0.295)		−0.377 (0.323)	
Education			0.469		0.020		0.001
College	881 (90.5)	0.677 (0.695)		0.775 (0.288)		−0.397 (0.320)	
High school	93 (9.5)	0.731 (0.677)		0.864 (0.353)		−0.278 (0.370)	

Abbreviations: LDL-C: low-density lipoprotein cholesterol, and BMI: body mass index; *p* value determined by *t*-test. * Median (IQR): median and interquartile range of urinary phthalate metabolites.

**Table 2:** Univariable linear regression of urinary phthalate metabolites and time-domain heart rate variability (HRV) analysis parameters.

HRV	Log Mean RRI		Log SDNN (ms)		Log RMSSD (ms)		Log pNN50 (%)		Log HRV Triangular Index		Log TINN (ms)	
	β (SE)	*p*	β (SE)	*p*	β (SE)	*p*	β (SE)	*p*	β (SE)	*p*	β (SE)	*p*
MEHP	0.001 (0.009)	0.786	−0.012 (0.009)	0.182	−0.005 (0.012)	0.645	−0.021 (0.032)	0.507	−0.008 (0.008)	0.299	−0.011 (0.010)	0.266
MEHHP	0.006 (0.007)	0.349	−0.032 (0.020)	0.121	−0.003 (0.028)	0.903	0.039 (0.074)	0.597	−0.041 (0.018)	0.025	−0.066 (0.023)	0.005
MEOHP	0.004 (0.006)	0.495	−0.034 (0.019)	0.071	−0.008 (0.026)	0.751	0.035 (0.068)	0.608	−0.040 (0.017)	0.016	−0.051 (0.021)	0.017
ΣDEHP	0.059 (0.235)	0.801	−0.132 (0.077)	0.089	−0.041 (0.057)	0.468	−0.014 (0.023)	0.531	−0.141 (0.087)	0.105	−0.138 (0.069)	0.044

MEHP, MEHHP, and MEOHP: log transformations with creatinine-adjusted (μg/g creatinine). ΣDEHP: log-transformed and creatinine-corrected molecular summation of MEHP, MEHHP, and MEOHP. Abbreviations: MEHP: mono-(2-ethylhexyl) phthalate, MEHHP: mono (2-ethyl-5-hydroxyhexyl) phthalate, MEOHP: mono-(2-ethyl-5-oxohexyl) phthalate, RRI: R–R intervals, SDNN: Standard deviation of NN intervals, RMSSD: root mean square of successive RR interval differences, pNN50: percentage of successive RR intervals that differ by more than 50 ms, and TINN: baseline width of the RR interval histogram, Beta: beta coefficient, and SE: standard error.

**Table 3:** Univariable linear regression of urinary phthalate metabolites and frequency-domain heart rate variability (HRV) analysis parameters.

	Log VLF (0.00–0.04 Hz)		Log LF (0.04–0.15 Hz)		Log HF (0.15–0.4 Hz)		Log LF/HF	
	β (SE)	*p*-Value	β (SE)	*p*-Value	β (SE)	*p*-Value	β (SE)	*p*-Value
MEHP	−0.160 (0.022)	0.460	−0.027 (0.021)	0.198	−0.016 (0.024)	0.512	−0.008 (0.020)	0.688
MEHHP	−0.154 (0.051)	0.003	−0.112 (0.049)	0.022	−0.004 (0.056)	0.941	−0.112 (0.047)	0.018
MEOHP	−0.101 (0.047)	0.032	−0.134 (0.044)	0.003	−0.019 (0.051)	0.715	0.121 (0.043)	0.005
ΣDEHP	−0.046 (0.031)	0.137	−0.062 (0.032)	0.058	−0.027 (0.028)	0.342	−0.026 (0.033)	0.435

MEHP, MEHHP, and MEOHP: log transformations with creatinine-adjusted (μg/g creatinine). ΣDEHP: log-transformed and creatinine-corrected molecular summation of MEHP, MEHHP, and MEOHP. Abbreviations: MEHP: mono-(2-ethylhexyl) phthalate, MEHHP: mono (2-ethyl-5-hydroxyhexyl) phthalate, MEOHP: mono-(2-ethyl-5-oxohexyl) phthalate, Beta: beta coefficient, and SE: standard error, VLF: very low frequency, LF: low frequency, and HF: high frequency.

**Table 4:** Multivariable linear regression of urinary phthalate metabolites and time-domain HRV analysis parameters.

	Log HRV Triangular Index		Log TINN (ms)	
	β (95% CI)	*p*-Value	β (95% CI)	*p*-Value
MEHP	−0.001 (−0.015~0.014)	0.918	−0.008 (−0.270~0.012)	0.432
MEHHP	−0.019 (−0.053~0.016)	0.290	−0.057 (−0.102~-0.011)	0.016
MEOHP	−0.017 (−0.048~0.015)	0.310	−0.040 (−0.082~0.003)	0.067
ΣDEHP	−0.005 (−0.027~0.017)	0.652	−0.026 (−0.056~0.004)	0.091

Adjusted Model: linear regression adjusted for age, sex, BMI z-score, systolic blood pressure, fasting sugar, and LDL-C. MEHP, MEHHP, and MEOHP: log transformations with creatinine-adjusted (μg/g creatinine). ΣDEHP: log-transformed and creatinine-corrected molecular summation of MEHP, MEHHP, and MEOHP. Abbreviations: MEHP, MEHHP, MEOHP, HRV: see Table 3, TINN: baseline width of the RR interval histogram, Beta: beta coefficient, and CI: confidence interval.

**Table 5:** Multivariable linear regression of urinary phthalate metabolites and frequency-domain HRV analysis parameters.

	Log VLF (0.00–0.04 Hz)		Log LF (0.04–0.15 Hz)		Log HF (0.15–0.4 Hz)		Log LF/HF	
	β (95% CI)	*p*-Value	β (95% CI)	*p*-Value	β (95% CI)	*p*-Value	β (95% CI)	*p*-Value
MEHP	−0.004 (−0.047~0.038)	0.839	−0.005 (−0.043~0.034)	0.813	0.010 (−0.033~0.052)	0.651	−0.011 (−0.049~0.027)	0.573
MEHHP	−0.010 (−0.210~0.010)	0.031	−0.041 (−0.133~0.050)	0.373	0.067 (−0.034~0.167)	0.194	−0.110 (−0.200~-0.020)	0.016
MEOHP	−0.049 (−0.141~0.043)	0.297	−0.053 (−0.137~0.031)	0.213	0.043 (−0.049~0.136)	0.359	−0.101 (−0.184~-0.019)	0.001
ΣDEHP	−0.026 (−0.090~0.039)	0.436	−0.017 (−0.076~0.042)	0.567	0.016 (−0.049~0.081)	0.628	−0.030 (−0.088~0.028)	0.304

Adjusted Model: linear regression adjusted for age, sex, BMI z-score, systolic blood pressure, fasting glucose, and LDL-C. MEHP, MEHHP, and MEOHP: log transformations with creatinine-adjusted (μg/g creatinine). ΣDEHP: log-transformed and creatinine-corrected molecular summation of MEHP, MEHHP, and MEOHP. Abbreviations: MEHP: mono-(2-ethylhexyl) phthalate, MEHHP: mono (2-ethyl-5-hydroxyhexyl) phthalate, MEOHP: mono-(2-ethyl-5-oxohexyl) phthalate, Beta: beta coefficient, CI: confidence interval, VLF: very low frequency, LF: low frequency, and HF: high frequency.

**Table 6:** One-way analysis of variance for urinary phthalate metabolites and the autonomic balance, LF/HF ratio.

Log MEHHP	Log LF/HF			Log MEOHP	Log LF/HF		
	Mean	SD	*p*-Value for Linear Trend		Mean	SD	*p*-Value for Linear Trend
1st quartile (<0.589)	0.137	0.446	0.014	1st quartile (<−0.592)	0.143	0.450	0.001
2nd quartile (<0.774)	0.112	0.386		2nd quartile (<−0.395)	0.123	0.411	
3rd quartile (<0.965)	0.054	0.482		3rd quartile (<−0.185)	0.084	0.437	
4th quartile (≥0.965)	0.088	0.438		4th quartile (≥−0.185)	0.089	0.452	

Log MEHHP and Log MEOHP: log transformations with creatinine-adjusted (μg/g creatinine). Abbreviations: MEHHP: mono (2-ethyl-5-hydroxyhexyl) phthalate, MEOHP: mono-(2-ethyl-5-oxohexyl) phthalate, LF: low frequency, HF: high frequency; p for testing trend of means by one-way analysis of variance (ANOVA).

## Supplementary Materials

The following are available online at https://www.mdpi.com/article/10.3390/toxics9120351/s1, Table S1: Linear regression coefficients of cardiovascular disease risk factors and time-domain HRV analysis, Table S2: Linear regression coefficients of cardiovascular disease risk factors and frequency-domain HRV analysis.
