# Supplementary material for: Association between Levels of Urine Di-(2-ethylhexyl)phthalate Metabolites and Heart Rate Variability in Young Adults
Source: Toxics. 2021 Dec 12;9(12):351. doi: 10.3390/toxics9120351 (PMC8709404; doi:10.3390/toxics9120351)
Supplement: Supplementary file 1 [file toxics-09-00351-s001.zip › toxics-1472762-supplementary.pdf]

toxics

Article

Supplementary Materials: Association between Levels of Urine Di-(2-ethylhexyl)phthalate Metabolites and Heart Rate Variability in Young Adults

Ching-Way Chen, Shu-Yu Tang, Jin-Shiang Hwang, Chang-Chuan Chan, Cheng-Chih Hsu, Chien-Yu Lin and Ta-Chen Su

Table S1. Linear regression coefficients of cardiovascular disease risk factors and time-domain HRV analysis.

	Mean RRI		SDNN		RMSSD		pNN50		Triangular index		TINN	
	**Beta**	***p*-value**	**Beta**	***p*-value**	**Beta**	***p*-value**	**Beta**	***p*-value**	**Beta**	***p*-value**	**Beta**	***p*-value**
Age, (yrs)	−0.001	0.050*	−0.010	0.001*	−0.017	0.001*	−0.037	0.001*	−0.009	0.001*	−0.007	0.001*
Men	0.019	0.001*	0.058	0.001*	0.004	0.821	−0.055	0.202	0.027	0.014*	−0.003	0.823
BMI Z score	−0.007	0.001*	−0.004	0.528	−0.040	0.001*	−0.107	0.001*	−0.005	0.317	0.009	0.189
SBP, (mmHg)	−0.001	0.001*	−0.001	0.001*	−0.005	0.001*	−0.011	0.001*	−0.001	0.001*	0.001	0.083
DBP, (mmHg)	−0.001	0.001*	−0.002	0.001*	−0.006	0.001*	−0.014	0.001*	−0.002	0.001*	0.000	0.513
Glucose, (mg/dL)	−0.007	0.001*	−0.021	0.001*	−0.035	0.001*	−0.040	0.106	−0.016	0.001*	−0.010	0.123
HbA1C (%)	−0.014	0.001*	−0.036	0.001*	−0.075	0.001*	−0.141	0.001*	−0.025	0.002*	−0.009	0.379
Cholesterol, (mg/dL)	−0.006	0.001*	−0.010	0.069	−0.026	0.001*	−0.049	0.001*	−0.005	0.273	0.009	0.158
LDL-C, (mg/dL)	−0.005	0.010	−0.007	0.270	−0.030	0.001	−0.076	0.001	−0.006	0.292	0.009	0.237
Creatinine, (mg/ dL)	0.002	0.544	−0.017	0.096	−0.029	0.042	0.003	0.981	−0.019	0.045	−0.025	0.033
Smoker	0.012	0.016	0.029	0.067	0.012	0.561	0.037	0.517	0.005	0.7310	−0.002	0.924
Education	0.004	0.537	0.013	0.526	0.014	0.613	−0.105	0.171	0.015	0.402	−0.020	0.389

Abbreviations: BMI: body mass index, SBP: systolic blood pressure; DBP: diastolic blood pressure; Glucose: fasting blood glucose; HbA1C: glycated hemoglobin, %; LDL: low-density lipoprotein, Mean RRI: log-transformed mean RR index, SDNN: log-transformed standard deviation of NN intervals, RMSSD: log-transformed root mean square of successive RR interval differences, pNN50: log-transformed percentage of successive RR intervals that differ by more than 50 ms, TINN: log-transformed baseline width of the RR interval histogram, Beta: beta coefficient.* p value < 0.05.

Table S2. Linear regression coefficients of cardiovascular disease risk factors and frequency-domain HRV analysis.

	**Log VLF (0.00–0.04 Hz)**		**Log LF (0.04–0.15 Hz)**		**Log HF (0.15–0.4 Hz)**		**Log LF/HF Ratio**	
	**Beta**	***p*-value**	**Beta**	***p*-value**	**Beta**	***p*-value**	**Beta**	***p*-value**
Age, yrs	−0.008	0.019*	−0.023	0.001*	−0.034	0.001*	0.012*	0.001*
Men	0.161	0.001*	0.163	0.001*	−0.042	0.210	0.217	0.001*
BMI Z score	0.047	0.002*	−0.003	0.808	−0.095	0.001*	0.095	0.001*
SBP, mmHg	0.001	0.641	−0.003	0.008*	−0.011	0.001*	0.008*	0.001*
DBP, mmHg	−0.002	0.126	−0.005	0.001*	−0.013	0.001*	0.007*	0.001*
Glucose, mg/dL	−0.026	0.050*	−0.031	0.017*	−0.082	0.001*	0.051	0.001*
HbA1C, %	−0.015	0.500	−0.068	0.002*	−0.165	0.001*	0.095	0.001*
Cholesterol, mg/dL	−0.008	0.575	−0.005	0.703	−0.048	0.002*	0.044	0.001*
LDL-C, mg/dL	0.020	0.217	0.011	0.490	−0.062	0.001*	0.074	0.001*
Creatinine, mg/ dL	−0.018	0.484	−0.035	0.160	−0.071	0.012*	0.037*	0.122
Smoker	0.042	0.283	0.054	0.145	−0.006	0.897	0.057	0.119
Education	0.040	0.443	0.064	0.190	0.030	0.595	0.002*	0.971

Abbreviations: BMI: body mass index; SBP: systolic blood pressure, DBP: diastolic blood pressure, Glucose: fasting blood glucose, HbA1C: glycated hemoglobin, LDL-C: low density lipoprotein cholesterol.* p value < 0.05.
